# The feasibility of resistance training versus aerobic exercise in a rehabilitation setting for people living with psychotic disorders: A randomised controlled trial

**DOI:** 10.1177/00048674251361681

**Published:** 2025-11-11

**Authors:** Nicole Korman, Robert Stanton, Mike Trott, Brendon Stubbs, Andrea Baker, Cassandra Butler, Dan Siskind, Simon Rosenbaum, Joseph Firth, Rebecca Martland, Talia McIntosh, Nicola Warren, Edward Heffernan, Frances Dark, Justin Chapman

**Affiliations:** 1 Metro South Addiction and Mental Health Service, Metro South Health, Brisbane, QLD, Australia; 2 Queensland Centre for Mental Health Research, Wacol, QLD, Australia; 3 Department of Psychiatry, Faculty of Health, Medicine and Behavioural Sciences, University of Queensland, Brisbane, QLD, Australia; 4 School of Health, Medical, and Applied Sciences, Central Queensland University, Rockhampton, QLD, Australia; 5 Department of Psychological Medicine, Institute of Psychiatry, Psychology and Neuroscience, King’s College London, London, UK; 6 School of Health, University of the Sunshine Coast, Sunshine Coast, QLD, Australia; 7 Nutrition, Exercise and Social Equity Research Group, Discipline of Psychiatry and Mental Health, School of Clinical Medicine, University of New South Wales, Sydney, NSW, Australia; 8 Division of Psychology and Mental Health, Manchester Academic Health Science Centre, University of Manchester, Manchester, UK; 9 Queensland Forensic Mental Health Service, Queensland Health, Brisbane, QLD, Australia; 10 Forensic Mental Health Group, Queensland Centre for Mental Health Research, Wacol, QLD, Australia; 11 School of Pharmacy and Medical Sciences, Centre for Mental Health, Griffith University, Brisbane, QLD, Australia

**Keywords:** Resistance training, psychotic disorders, schizophrenia, exercise, psychiatric rehabilitation

## Abstract

**Objectives:**

People with psychotic disorders face significant functional impairments, high levels of disability, multimorbidity and physical health challenges. Despite unique health benefits, resistance training remains underexplored in this population and rarely implemented in real-world mental health settings.

**Methods:**

This randomised controlled trial comparing resistance training with aerobic interval training in people with psychotic disorders accessing psychiatric rehabilitation. Supervised exercise sessions by exercise physiologists were conducted 3 times per week over 8 weeks. Primary outcomes were feasibility, acceptability and adverse events. Secondary outcomes were psychiatric symptoms, global and physical functioning and the effect of randomisation to exercise type on participation rates.

**Results:**

In total, 54 participants (median age 31 years, 71.6% male, 75% diagnosed with schizophrenia/schizoaffective disorder, 55.5% with ⩾3 health conditions) were enrolled. Resistance training met predetermined feasibility and acceptability thresholds and showed comparable results to aerobic interval training with no significant exercise-related adverse events. Within-group analysis revealed significant increases in muscle strength following resistance training. Post-intervention, resistance training participants reported more total weekly minutes of physical activity compared to aerobic interval training, though no other significant between-group differences were observed. Randomisation to exercise type did not influence participation.

**Conclusion:**

In conclusion, resistance training was feasible and acceptable to people with psychotic disorders, with no serious adverse events and comparable to aerobic interval training. Resistance training was successfully implemented in rehabilitation settings with promising improvements in muscle strength and self-reported physical activity. In future, larger longer-term trials comparing resistance training with aerobic interval training, and in comparison with other psychosocial therapies are warranted. Further exploration of participant preference for exercise type on outcomes is recommended.

## Introduction

People living with psychotic disorders, including schizophrenia and related psychoses, face significant functional impairments (Correll and Schooler, 2020), contributing to high rates of disability (Ferrari et al., 2022). In this population, functional impairments (Nygård et al., 2019, 2021; Vancampfort et al., 2012) are associated with very low cardiorespiratory fitness (Heggelund et al., 2020; Vancampfort et al., 2015a) and muscular weakness (Nygård et al., 2019; Viertiö et al., 2009) with these markers of physical functioning of a severity that resembles premature ageing (Pearson et al., 2023). Contributing factors include high levels of sedentary behaviour (Stubbs et al., 2016) and low engagement in moderate to vigorous physical activity (PA) (Vancampfort et al., 2017) beginning early in the illness (Lee et al., 2013). This is further influenced by complex factors such as medication side effects, motivational deficits, stress and low mood, obesity, pain and low social support to exercise (Arnautovska et al., 2022).

Cardiorespiratory fitness is necessary for exertional tasks related to activities of daily living (Sebastião et al., 2019), while muscular strength is vital for locomotion, falls prevention and lifting and carrying tasks (Aagaard et al., 2010; Salem et al., 2000; Suetta et al., 2004). Resistance training (RT) (also known as weight/strength training) has promising health benefits independent of aerobic training (Brellenthin et al., 2021) that may restore and enhance physical functioning in the general population (Prado et al., 2018; Rizzoli et al., 2013) and support activities of daily living (ADLs) (Mangione et al., 2010).

While RT has shown to have mental health benefits, such as reduced depression (Gordon et al., 2018; Stanton et al., 2013) and anxiety (Cassilhas et al., 2010; Gordon et al., 2017, 2021), its effectiveness on psychotic disorders remains under-researched (Stubbs et al., 2018). Despite promising improvements in strength from several studies (Gallardo-Gómez et al., 2023), there are inconsistent outcomes for global and psychological functioning (Keller-Varady et al., 2018; Korman et al., 2023; Sabe et al., 2020) and its feasibility in real-world mental health settings remains underexplored. In addition, few studies have directly compared RT with other exercise types, such as aerobic training (García-Garcés et al., 2021a).

Psychiatric rehabilitation services are a type of specialist mental health service which aim to improve independent functioning across a range of health domains (Killaspy et al., 2005; Wolfson et al., 2019). In Australia, most individuals accessing these services will be diagnosed with schizophrenia or a related disorder and have complex physical and mental health needs and substantially lower functioning than people with schizophrenia living in the community (Meehan et al., 2017; Parker et al., 2019). The holistic approach, longer duration of stay and focus on functioning (Wolfson et al., 2019) provide an ideal service type within which to integrate exercise interventions (Daumit et al., 2011). However, few published studies have explored the implementation of exercise in these settings, and those that have evaluated aerobic exercise (Daumit et al., 2013; Forsberg et al., 2008; Korman et al., 2018), with none focusing on RT. This study aimed to evaluate the feasibility, acceptability and safety of RT in people with psychotic disorders within rehabilitation settings in comparison with aerobic exercise. Secondary aims include comparing RT with aerobic interval training (AIT) on global and physical functioning and psychiatric symptoms. Given this was a novel exploratory feasibility study, no a priori hypothesis was proposed.

## Materials and methods

### Study design

This was a single-blind, two-arm pragmatic feasibility randomised controlled trial (RCT). Ethical approval was provided by the Metro South Health Human Research Ethics Committee (HREC/20267647); the trial was prospectively registered with the Australian and New Zealand Clinical Trials Registry (ANZCTR: ACTRN12620001309976). The trial was conducted in accordance with the Declaration of Helsinki (World Medical Association, 2024) and followed CONSORT guidelines for pilot feasibility trials (Eldridge et al., 2016). In light of findings from a previous uncontrolled study in this setting assessing feasibility and acceptability of a combined exercise and diet intervention (*n* = 42) (Korman et al., 2020), and based on budgetry constraints, anticipated recruitment and retention, we estimated that 25 per group may be sufficient to evaluate feasibility (retention, participation and acceptability) (Teresi et al., 2022).

### Participants

Participants from three psychiatric residential rehabilitation units were referred by mental health staff and screened by the research team between January 2021 and October 2023. These units provide accommodation and 24-hour multidisciplinary mental health support, admitting residents deemed low imminent risk to self or others (Queensland Health, 2015). Participants were eligible if they were (1) aged 18–64 years; (2) diagnosed with a *DSM*-5 psychotic spectrum disorder (e.g. schizophrenia, schizoaffective disorder) confirmed by the treating psychiatrist; (3) likely to reside in the unit for the study duration; (4) cleared for exercise by an accredited exercise physiologist (AEP) or general practitioner; and (5) provided informed consent. Participants were excluded if they (1) were pregnant; (2) had a comorbid eating disorder contraindicating exercise determined by the treating psychiatrist; (3) had substance use issues that interfered with rehabilitation; or (4) demonstrated inability to understand the study or follow study instructions.

#### Consent

Researchers experienced in obtaining consenting from individuals with psychosis explained risks and study restrictions. For the duration of the study, participants were asked not to engage in an exercise type other than the one they were allocated, outside of supervised study sessions. Those who declined could continue accessing existing exercise services in the rehabilitation unit.

### Procedures

#### Randomisation

Participants were randomised 1:1 to RT or AIT using a computer-generated sequence with block randomisation (block size: four). Allocation codes were prepared by an independent biostatistician and provided in sealed envelopes. Participants were not blinded to their allocation.

#### Interventions

Both interventions involved moderate-intensity exercise three times weekly for 30 minutes over 8 weeks (24 sessions): these parameters are consistent with evidence supporting physical and mental health benefits in individuals with schizophrenia (Firth et al., 2015). Sessions were supervised by AEPs experienced in mental health settings, with intensity monitored using the Borg Category Ratio 10 scale for perceived exertion (Borg, 1982). AEPs were trained in the protocol (see Supplemental Appendix 2 for full protocol). Programme records were reviewed by the research team regularly to maintain consistency with protocol to ensure fidelity. Interventions were integrated into the multidisciplinary rehabilitation model (Dark et al., 2018; Vita et al., 2021) to allow flexibility in session scheduling (Korman et al., 2020; Korman et al., 2018), and the provision of behavioural support strategies recommended when engaging individuals with severe mental illness (SMI) in exercise (Arnautovska et al., 2022; Stubbs et al., 2018).

The intervention structure and progression is included in Tables 1 and 2, and described using the Consensus on Exercise Reporting Template (Slade et al., 2016) (Supplemental Appendix 3). An example of an RT prescription and clinical photographs are found in Supplemental Appendix 4. The Behaviour Change Wheel and COM-B model (Michie et al., 2011) and Theoretical Domains Framework (TDF) (Atkins et al., 2017) were used to map intervention components (Supplemental Appendix 5).

**Table 1.** Intervention structure.

	Resistance training (RT)	Aerobic interval training (AIT)
Warm up	5 minutes aerobic RPE (1–2), +5 minutes stretching	10 minutes aerobic (RPE 1–2)
Equipment	A variety of standard resistance training equipment (hand weights, kettle bells, resistance bands, squat rack, chest press bar and floor mats appropriate for small clinical gym settings	Treadmill, elliptical, rowing machine, seated bicycle
Prescription	6–8 main muscle groups, arms, shoulders, back, core, lower limbs Upper and lower body exercises were alternated between sessions to facilitate recovery. 3–4 sets of 8–12 repetitions, ~ 90 sec passive rest interval between sets until active exercise session duration reached The content and weights of each session was recorded using standard protocols	Any aerobic activity that aims to increase cardiorespiratory fitness using preferred gym equipment and an interval training structure 4 minutes active aerobic exercise, alternating with 3 minutes active recovery (reduced intensity of the active recovery interval, RPE 1–2) until session duration reached The equipment and active intervals were recorded each session using standard protocols
Cool down	5 minutes stretch	5 minutes stretch

RT: resistance training; AIT: aerobic interval training; RPE: rate of perceived exertion.

**Table 2.** Progression.

Time point	RPE	Duration of active exercise	Prescription
Session 1	2–3 (RT – 40% 1–RM)	30 minutes	Familiarisation with equipment and correct exercise technique.
Week 1–3	2–3	30 minutes	Prescription reviewed weekly: progression individualised and could be regressed/progressed per capacity and exercise history. RT progression through increases in load. AIT progression through increases in intensity.
Week 4	4 (RT – 40–70%)	40 minutes	Progression individualised and could be regressed/progressed per response
Week 5–8	⩾4	45–55 minutes	Progression individualised and could be regressed/progressed per response

RPE: rate of perceived exertion; RM: repetition max; RT: resistance training; AIT: aerobic interval training; mins: minutes.

### Data collection

Baseline assessments included socio-demographics, clinical information (e.g. diagnoses, medication use, converted to olanzapine equivalents) (Taylor et al., 2021), and an exercise preferences questionnaire (Firth et al., 2016b; Ussher et al., 2007). Participants indicated their preference for RT, AIT, or neither before randomisation (Rosenbaum et al., 2016). Incentives were a $40 gift card provided upon completion of each assessment point (baseline or 8-week endpoint). Instead of gift cards, participants could choose a Garmin VivoFit watch, provided following completion of the 8-week endpoint assessment. Participants were invited to complete post-intervention assessments regardless of discontinuation from the intervention. Trained research nurses blind to participant allocation completed research measures with participants. Study participants were recruited from December 2020, with follow up completed in December 2023.

#### Primary outcome measures

*Feasibility*: Attendance was recorded when participants completed over 50% of a planned exercise session. Feasibility was defined a priori as attending ⩾65% of sessions (16 out of 24) and completion of the eight week intervention by ⩾70% of participants per group, reflecting benchmarks from similar studies (Bartels, 2015; Jerome et al., 2017).

*Acceptability*: Participant acceptability was assessed post-intervention using a self-report questionnaire based on the Theoretical Framework of Acceptability (Sekhon et al., 2017), consisting of 14 items adapted from previous questionnaires used with people with mental illnesses (Chapman et al., 2015; Korman et al., 2020). Responses were on 14 items (e.g. enjoyment, mood, confidence to continue this type of exercising alone) measured on a 5-point scale of agreement (ranging from *strongly disagree* to *strongly agree*) (Supplemental Appendix 6). An aggregate acceptability score was calculated by summing item responses with reverse scoring for negative statements (ranging from −28 to 28). Each item was considered acceptable if ⩾65% of participants endorsed the item (Chapman et al., 2016).

*Adverse events (AEs)*: AEs were monitored by the AEP at every session via verbal check-ins and documented according to Good Clinical Practice guidelines (International Council for Harmonisation (ICH), 2018) (Supplemental Appendix 2: Intervention protocol for comprehensive details)

#### Secondary outcome measures

All participants were assessed at baseline and post-intervention (8 weeks) (Supplemental Appendix 1: Schedule of visits) for the following measures (Supplemental Appendix 2: Intervention protocol for full details).

##### Global functioning

*World Health Organization Disability Adjusted Scale (WHODAS) 2.0*: is a reliable and valid 36-item questionnaire administered by an interviewer to assess the health and disability in all adult populations across different cultures; covering six domains of functioning including cognition, mobility, self-care, getting along, life activities and participation (ÜStÜN et al., 2010).

##### Psychiatric symptoms

*Brief Psychiatric Rating Scale (BPRS)*: was developed for assessment of change across a broad range of psychiatric symptoms, is brief, reliable, sensitive and has been used extensively across a variety of settings and patients (Crippa et al., 2001; Ligon and Thyer, 2000).

*Scale Assessment of Negative Symptoms (SANS)*: is a reliable and valid scale, using the Interview Guide for Assessment of Negative Symptoms (IG-SANS) (Alonso et al., 2008; Lyne et al., 2013).

##### Physical functioning

*Simple Physical Activity Questionnaire (SIMPAQ)*: is a researcher-administered self-report PA questionnaire assessing time engaging in PA and sedentary behaviour in the previous week. The SIMPAQ has been internationally validated and is correlated with objective PA in people with SMI (Rosenbaum et al., 2020).

*Six-minute walk test (6MWT)*: is a submaximal test to assess functional capacity that is commonly used for adults with mental illness as a proxy measure of fitness (Bernard et al., 2015).

*Thirty*-*second sit-to-stand-to-sit test (STS)*: evaluates lower body strength by counting sit-to-stand cycles in 30 seconds (participants were asked to stand up from a seated position and sit back down as many times as possible without using their arms in 30 seconds). This test has been shown to correlate well with a 1-RM (one repetition maximum) leg-press test in community-dwelling older adults (*r* > 0.7) and has good test–retest reliability (Jones et al., 1999).

*Grip strength (maximal isometric handgrip)*: measures upper body strength using a dynamometer. Maximal isometric handgrip is considered suitable for a variety of clinical settings and provides a simple and valid proxy of upper body muscular strength (Roberts et al., 2011).

*Push-up test*: As Many Rounds As Possible (AMRAP) of push-ups completed in 1 minute, using modified or full push-ups (knees, full body push up, or against a wall incline) (Herron et al., 2016).

*Muscular strength*: Bench press and squat 12RM (kg) were assessed by the AEP pre and post the intervention; only assessed in the RT group due to complexity of the testing.

### Analysis

Statistical analysis was conducted using SPSS Statistics 24 and R. Continuous variables were described using means and standard deviations (SDs), with normality tested using the Shapiro-Wilks test and Q-Q plot inspection. Demographic and clinical variable at baseline, and statistics on feasibility, acceptability and AEs were compared between groups using independent *t*-tests or Mann–Whitney *U* tests; categorical data was compared through chi-square analysis. Secondary analyses were conducted using an intention-to-treat approach, implementing a Linear Mixed Effects Model in R (Bates et al., 2015; R Core Team, 2024) with fixed effects for treatment group, time, time*treatment group interaction, and covariates including age, gender, exercise participation, antipsychotic medication use and random effects for individual participants. Post hoc Bonferroni analyses were performed to explore any significant group or time differences. A one-way analysis of variance (ANOVA) was employed to assess exercise participation rates across different exercise preference conditions. This multi-faceted statistical approach ensured a rigorous, detailed, and reliable examination of the study’s outcomes, allowing for robust interpretation of the research findings across various analytical dimensions.

## Results

### Participants

**Figure 1.**
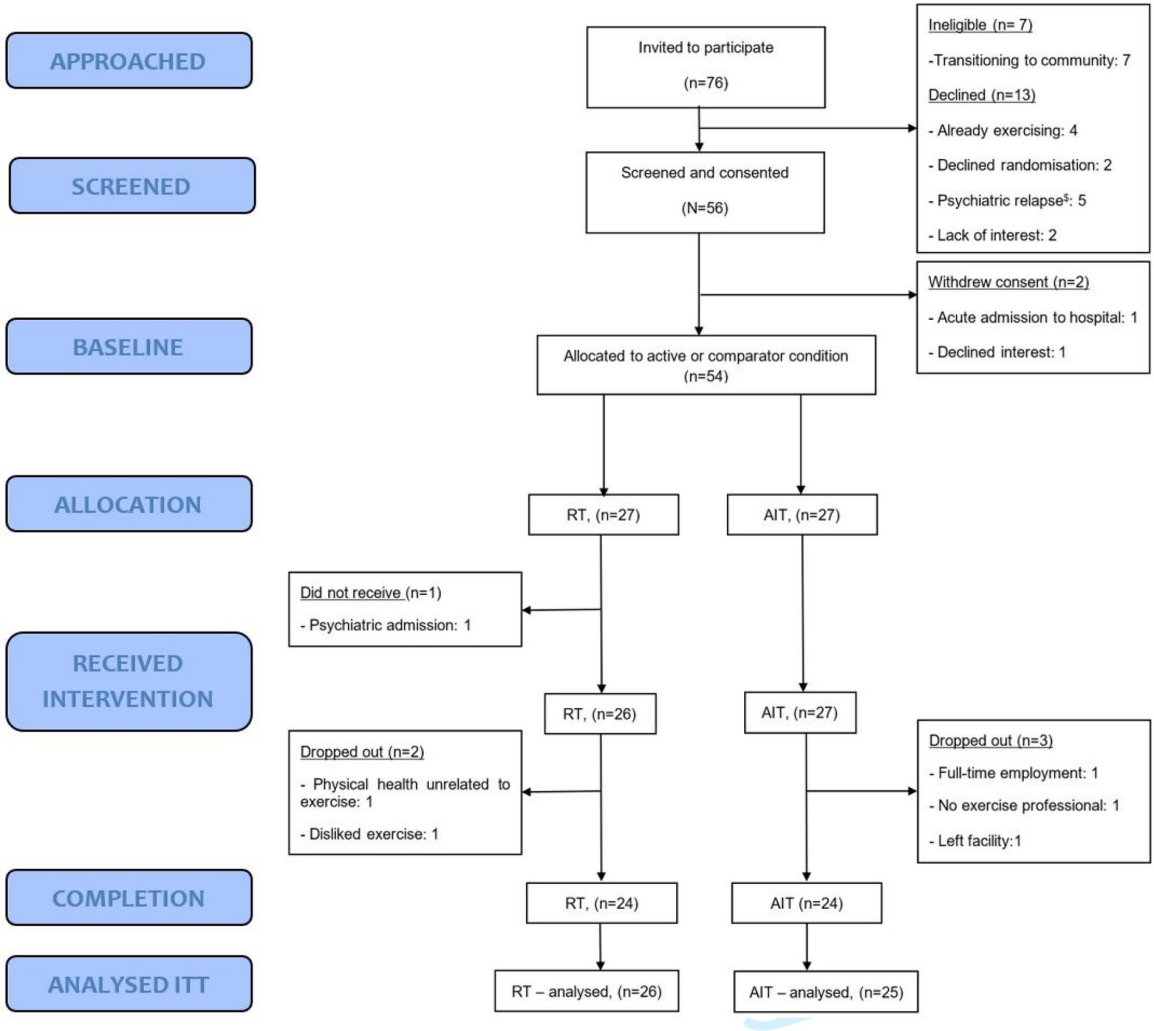
CONSORT diagram. ^$^Psychiatric relapse determined by treating psychiatrist.

Of the 76 referred individuals, 71% (54) consented; reasons for non-participation are in Figure 1 with only two declining randomisation to exercise type. One randomised participant could not complete the intervention due to COVID-related staffing issues.

Participant demographics are summarised in Table 3. The median age was 31 years, with 71.6% male and 75% diagnosed with schizophrenia or schizoaffective disorder. Most participants (55.5%) had three or more health conditions, and 62.2% were prescribed metabolically unfavourable antipsychotics (e.g. olanzapine, clozapine). The cohort’s mean body mass index (BMI) was 32.3 (SD 7.7), placing them in the obese class I category (Department of Health and Aged Care, 2021). Although two-thirds of participants self-reported engaging in aerobic or resistance exercise within the prior 3 months, baseline functional capacity and strength were comparable to norms for individuals aged 80–90 years (Tveter et al., 2014).

**Table 3.** Baseline characteristics.

Demographics	Total, *n* = 53	RT, *n* = 26	AIT, *n* = 27
Age^a^ (in years), median [IQR]	31 [12]	33.5 [12]	28 [11]
Sex, male, *n* (%)	38 (71.6%)	21 (80.8%)	17 (63%)
Diagnosis: *n* (%)			
Schizophrenia	32 (60.3%)	19 (73.1%)	13 (48%)
Schizoaffective disorder	8 (15%)	3 (11.5%)	5 (18.5%)
BPAD with psychosis	5 (9.4%)	2 (7.7%)	3 (11.1%)
Major depression + psychosis	3 (5.6%)	1 (3.8%)	2 (7.4%)
Drug induced psychosis	1 (1.8%)	0	1 (3.7%)
Psychotic disorder NOS	2 (3.7%)	0	2 (7.4%)
First Episode psychosis	2 (3.7%)	1 (3.8%)	1 (3.7%)
Education: *n* (%)			
Masters/higher degree	1 (1.8%)	1 (3.8%)	0
Bachelor degree	8 (15%)	8 (30.8%)	0
Trade	8 (15%)	1 (3.8%)	7 (25.9%)
Year 12	21 (39%)	9 (34.6%)	12 (44%)
Year 11	5 (9.4%)	2 (7.7%)	3 (11.1%)
Year 10	8 (15%)	3 (11.5%)	5 (18.5%)
Years 7–9	2 (3.7%)	2 (7.9%)	0
Employment: *n* (%)			
Part time	3 (5.6%)	1 (3.8%)	2 (7.4%)
Unemployed	50 (94.3%)	25 (96.2%)	25 (92.6%)
Receiving government income, *n* (%)	52 (94.3%)	26 (100%)	26 (96.3%)
Aboriginal/ Torres Straight Islander, *n* (%)	1 (1.9%)	1 (3.8%)	0
Duration in rehabilitation^a^ (in months), median [IQR]	4 [9]	4 [8]	4 [11]
Has substance use disorder, *n* (%)	5 (9.4%)	2 (7.7%)	3 (11.1%)
Duration illness^a^ (in years), median [IQR]^b^	5 [11.5]	8.5 [17]	3 [6.5]
Olanzapine/clozapine/both, *n* (%)	33 (62.2%)	17 (65.4%)	16 (59%)
Olanzapine equivalents (in mg), mean (SD)	25 (28.8)	31.3 (20)	26.6 (18)
Number of psychotropic medications^a^, median [IQR]	2 [1]	2 [1]	2 [1]
Total number of medications^a^, median [IQR]	4 [2]	3.5 [2]	4 [3]
Has cardiometabolic condition, *n* (%)	31 (58.4%)	16 (61.5%)	15(55.5%)
Total number of health conditions, mean (SD)	3 (2)	3 (2)	2.9 (2.1)
Multimorbidity (⩾3 health conditions), *n* (%)	29 (55%)	13 (50%)	16 (59.3%)
Number of musculoskeletal injuries at baseline, *n* (%)	9 (16.9%)	7 (26.9%)	2 (7.4%)
Smoking, yes, *n* (%)	20 (37.7%)	11 (42.3%)	9 (33.3%)
BMI, mean (SD)	32.3 (7.7)	32.4 (7.7)	31.6(7.9)
Waist circumference (in cm), mean (SD)	105.9 (18.7)	108.5 (17.7)	103.5 (19.7)
Baseline physical functioning:			
Function exercise capacity, mean (SD)			
Males	521 (69)	513(52)	531(85)
Females	460 (62.8)	435 (52)	472 (66.5)
Sit to stand, median [IQR]			
Males	11.79 [1.3]	11.8 [1.8]	11.7 [2.1]
Females^a^, median (IQR)	12 [4]	12 [5]	12 [6]

BMI: body mass index; NOS: not otherwise specified; BPAD: bipolar affective disorder; SD: standard deviation; *n*: number; median and IQR: interquartile range; mg: milligrams.

a Non-parametric statistics.

b *p*-value significant difference between groups.

RT and AIT groups were similar on baseline characteristics except for longer illness duration (8.5 years vs 3 years). Participants reported no group allocation preference 34% (*n* = 18), 24.5% (*n* = 13) preferred RT, and 41.5% (*n* = 22) preferred AIT (Supplemental Appendix 7: Table of Preferences, Barriers and Enablers).

### Primary outcomes

Feasibility, acceptability and AE data are presented in Table 4. Median participation rates were 23.5 (interquartile range [IQR]: 4) in RT and 22 (IQR 4) in AIT out of 24 sessions, with no significant group differences (*p* = 0.8). Intervention completion rates were 24/27 (88.8%) in both groups (*p* > 0.99).

**Table 4.** Comparison of feasibility and acceptability outcomes.

Outcome	Total	RT	AIT	Statistical test, RT v AIT	*p* value
Feasibility					
Completion of intervention, *n*/*N* (%)	48/54, 88.8%	24/27, 88.8%	24/27, 88.8%	Chi square Fisher’s exact	*p* => 0.99
Retention for analysis, *n*/*N* (%)	51/54, 94%	26/27, 96.2%	25/27, 92.5%	Chi square Fisher’s exact	*p* => 0.99
Participation rate, # sessions, median (IQR)	23 (4)	23.5 (4)	22 (4)	Mann Whitney *U* *U* = 364.5, *z* =−0.25	*p* = 0.8
Adverse events					
Serious adverse events, *n*, type	3, unrelated to exercise	1, psychiatric	2, *n* = 1 anaphylaxis *n* = 1 psychiatric	N/A	N/A
Exercise-related adverse events number	0	0	0	N/A	N/A
Dropouts due to exercise-related adverse events	0	0	0	N/A	N/A
Acceptability					
**Aggregate score: acceptability questionnaire**	12.8 (6.1)	12.6 (6)	13.1 (6.2)	Independent *t* test, *t*(49) = 0.31	*p* = 0.7
*Specific Items, n (%)*	Total sample, agree/strongly agree, *n* = 51	Resistance Training, agree/strongly agree, *n* = 26	Aerobic Interval Training, agree/strongly agree, *n* = 25	Fisher’s exact test	
Enjoyed the programme	48 (94.1%)	25 (96.2%)	23 (92%)	0.61	
Too difficult	8 (15.7%)	4 (15.4%)	4 (16%)	>0.99	
Helped with daily tasks	36 (76.2%)	18 (69.2%)	18 (72%)	>0.99	
Programme was fun	41 (80.4%)	20 (76.9%)	21 (84%)	0.72	
Wanted to stop	6 (11.8%)	3 (11.5%)	3 (12%)	>0.99	
Was challenging enough	42 (82.4%)	20 (76.9%)	22 (88%)	>0.99	
Keep this exercise going after the programme ends	40 (78.4%)	22 (84.6%)	18 (72%)	0.32	
Looked forward to the exercise programme	42 (82.4%)	20 (76.9%)	22 (88%)	0.47	
Three sessions per week too much	3 (5.9%)	2 (7.7%)	1 (4%)	>0.99	
Exercise helped my mental health	45 (88.2%)	22 (84.6%)	23 (92%)	0.67	
Wish I could have changed to a different type of exercise	6 (13.7%)	2 (7.7%)	5 (20%)	0.25	
The programme made positive changes to my body	38 (74.5%)	19 (73.1%)	19 (76%)	>0.99	
Confident to do this on my own after it ends	39 (76.5%)	20 (76.9%)	19 (76%)	>0.99	
Individual sessions improved my mood	44 (86.3%)	20 (76.9%)	24 (96%)	0.09	

RT: resistance training; AIT: aerobic interval training; *n*: number of participants; *N*: total number of participants in the group; #: number of sessions.

A priori acceptability criteria (>65% strongly agree/agree per item) were met across all 14 questionnaire items in both groups, with no significant differences except for a trend favouring AIT for mood improvement (*p* = 0.09). Aggregate acceptability scores were similar between RT (mean 12.8, SD 6.1) and AIT (mean 12.6, SD 6, *p* = 0.7) (Supplemental Appendix 8 for Raw Acceptability Data).

There were three serious AEs unrelated to the intervention (one in RT, two in AIT), but there were no exercise-related AEs in either group (Table 4). There were 39 instances of temporary mild muscular soreness or joint stiffness related to exercise that did not meet criteria for an AE: 34 in the RT group versus 5 in the AIT group. The majority were delayed onset muscle soreness (DOMS; *n* = 22) or joint stiffness (*n* = 7), which were treated conservatively with continued exercise (Hayes et al., 2023; Lewis et al., 2012). There were no dropouts due to exercise-related muscle soreness in either group; however, three participants rescheduled an RT session due to DOMS.

### Secondary outcomes

A significant group*time interaction was observed for total weekly PA (*p* = 0.04). Post hoc analysis revealed a 52.9 minute/week increase in RT (Cohen’s *d* = 0.36) and a 245 minute/week decrease in AIT (Cohen’s *d* = 1.7). There were no group*time differences for other secondary outcomes.

Within-group improvements were observed in the RT group for psychiatric symptoms (BPRS: LS mean change −6.05, *p* = 0.0034), negative symptoms (SANS: LS mean change −6.3, *p* = 0.04) and push-up performance (LS mean change + 1.5, *p* = 0.02). Strength significantly increased in RT for the 12RM bench press (49.8% increase, *p* < 0.001) and squat (34.1% increase, *p* < 0.001). No within-group changes were detected in AIT for any outcome (Table 5).

**Table 5.** Secondary outcomes.

Variable	Treatment group	LS mean – baseline (SE), *n*	LS mean – endpoint (SE), *n*	LS, mean change from baseline (SE), p value	Group*time interaction (SE)	*p* value, group/time interaction	Cohen’s *d*, group/time interaction
Global functioning (WHODAS 2.0)	RT	36.8 (3.8)	33.8 (3.8)	3.1 (3), *p* = 1.0	2.3 (4.3)	*p* = 0.6	−0.27
	AIT	37.2 (3.6)	331.8 (3.6)	5.3 (3.12), *p* = 0.54			
Psychiatric Symptoms (BPRS)	RT	59.4 (2.3)	53.1 (2.3)	6.05 (1.6)** *p* = 0.003	−3.5 (2.3)	*p* = 0.13	−0.67
	AIT	52.8 (2.1)	50.1 (2.1)	2.6 (1.6), *p* = 0.7			
Negative symptoms (SANS)	RT	28.6 (3.1)	22.3 (12.9)	6.3 (2.2)* *p* = 0.04	−4.9 (3.1)	*p* = 0.12	0.22
	AIT	28.6 (3.1)	22.3 (3.1)	1.3 (2.3) *p* = 1.00			
Functional exercise capacity (6MWT)	RT	483 (15.6)	467 (15.6)	−15.9 (9.9) *p* = 0.7	−8.6 (14)	*p* = 0.58	0.69
	AIT	495 (14.4)	488 (14.6)	−27.6 (10) *p* = 1.0			
Sit to stand	RT	12.1 (0.5)	12.4 (0.5)	−0.34 (0.5) *p* = 1.0	−0.3 (0.7)	*p* = 0.64	−0.67
	AIT	11.5 (0.5)	12.2 (0.5)	0.69 (0.5) *p* = 1.0			
Push ups	RT	21.9 (1.7)	26.4 (1.7)	4.5 (1.5)*, *p* = 0.02	2.4 (2.1)	*p* = 0.2	0.16
	AIT	24.8 (1.6)	26.9 (1.6)	2 (1.5), *p* = 1.0			
Hand grip (Right)	RT	68 (4.5)	65.2 (4.5)	−2.8 (3.1) *p* = 1.0	0.57 (4.45)	*p* = 0.89	0.06
	AIT	69.1 (4.2)	65.7 (4.2)	−3.3 (3.1) *p* = 1.0			
Hand grip (Left)	RT	66.9 (4.44)	61.3 (4.4)	−5 (2.9), *p* = 0.3	−6 (4)	*p* = 0.15	0.36
	AIT	63.9 (4.1)	64.3 (4.1)	0.4 (2.9) *p* = 1.0			
Total weekly mins of physical activity	RT	813 (93.5)	866 (93.5)	52.9 (109) *p* = 1.0	−297 (145)	*p* = 0.045*	−1.8
	AIT	785 (88.2)	540 (89.8)	−245 (103) se, *p* = 0.13			
Moderate/vigorous mins of physical activity	RT	124 (22.8)	139.9 (22.8)	15.9 (32.8)	0.55 (102)	*p* = 1.0	−1.2
	AIT	87.8 (23.3)	84.1 (23.3)	−3.7 (30.1)			
Strength (12RM) – squat	RT only	25.5 (3)	38.2 (3.9)	12.1 (2.3)*** *p* < 0.001	N/A	N/A	1.03
Strength (12RM) – bench press	RT only	25.2 (3)	33.8 (3.3)	8 (1.1)*** *p* < 0.001	N/A	N/A	1.4

LS: least squares; SE: standard error; CI: confidence interval; RT: RT; 12RM: 12 repetition maximal test; 6MWT: six-minute walk test; BPRS: Brief Psychiatric Rating Scale; SANS: Scale Assessment of Negative Symptoms; NA: not applicable; mins: minutes.

* *p* value of <0.05; **p value of <0.01; ***p value of <0.001.

Participation rates did not differ based on group allocation preferences (allocated to preference, non-preferred condition, or no preference; *p* = 0.35 (Table 6).

**Table 6.** Preferences for exercise and participation.

	Randomised to preference, mean (SD)	Did not get randomised to preference, mean (SD)	Did not have a preference, mean (SD)	One way ANOVA	*p* value
Participation (number of sessions)	22 (2.1)	22.5 (3.3)	20.7 (4.8)	*F*(2, 48) = 1.07	0.35

SD: standard deviation; ANOVA: analysis of variance.

### Real world trial considerations

The trial began recruitment 6 weeks prior to the COVID-19 pandemic and Australia’s first national lockdown. There was widespread disruption to all health service activities, including recruitment and delivery of this intervention, which impacted the overall expected duration of the trial. Advocacy for exercise as a mainstream intervention for people with SMI allowed the resumption of supervised individual exercise sessions with safe distancing and masks. However, the pandemic resulted in a service-wide restructuring and unexpected loss of temporary exercise professional roles, further impacting delivery of the intervention at two sites.

## Discussion

This pragmatic RCT demonstrated that RT was feasible and acceptable for individuals with psychotic disorders in a real-world community mental health setting, with no serious AEs. Our findings demonstrate that despite participants’ complex physical and mental health challenges, including high rates of multimorbidity, obesity and reduced physical functioning that resembles premature ageing, engagement in RT was high.

Feasibility and acceptability were high in both RT and AIT conditions. Completion of the intervention and participation rates for RT exceeded predetermined feasibility thresholds, with no difference between exercise types. These rates aligned with, or surpassed, the ranges for the small number of isolated RT trials in this population, which reported participation and completion between 53% and 93% (García-Garcés et al., 2021a; Heggelund et al., 2012; Maurus et al., 2020; Şenormancı et al., 2021; Silva et al., 2015), and rates of adherence for other exercise types for people with schizophrenia (Firth et al., 2015; Vancampfort et al., 2016).

RT three times per week was considered acceptable across a range of individual perceptions regarding exercise enjoyment, physical and functional improvements, and capacity to continue exercising alone. Interestingly, irrespective of allocation preference prior to randomisation, less than 10% expressed a desire to change RT at the end of the intervention. These findings align with a small number of studies indicating that people with psychotic disorders may prefer RT (Firth et al., 2016b; Korman et al., 2020; Subramaniapillai et al., 2016).

A large recent cross-sectional survey of people with SMI reported 58% had not engaged in recent muscle strengthening activities, with only 28% meeting the recommended guideline of two sessions per week. Weekly engagement in RT was significantly lower than aerobic exercise (Tew et al., 2023) reflecting trends in the general population which has been attributed to less emphasis in public campaigns around muscle strengthening recommendations (Bennie et al., 2020) and for people with SMI, reduced access to equipment, gym spaces and trained professionals. Despite its underrepresentation in research, and being previously highlighted as a priority area (Stubbs et al., 2018), RT may offer critical health benefits, including reduced mortality and lower risks of cancer, type 2 diabetes and cardiovascular disease (Giovannucci et al., 2021; Momma et al., 2022), which are all significant threats to the health of people with psychotic disorders (Firth et al., 2019). This study supports the feasibility of RT for people with psychotic disorders in line with public health recommendations (Bull et al., 2020) and emphasises the need to provide health literacy on its benefits to encourage broader participation (Bennie et al., 2020).

AEs are inconsistently reported in exercise trials for people with psychotic disorders (Stubbs et al., 2018), despite the importance of this outcome for implementation success (Greenhalgh et al., 2014). This study found no exercise-related AEs, though RT was associated with more instances of temporary DOMS. DOMS, common in untrained individuals, results from eccentric forces and unaccustomed loads, causing temporary muscle damage and inflammation. Proper management, including continued exercise and, if needed, anti-inflammatory medications, can aid recovery (Lewis et al., 2012). Musculoskeletal pain has been identified as a barrier to exercise in people with schizophrenia (Vancampfort et al., 2011) hence addressing expectations about DOMS proactively (Hurst et al., 2022) likely boosted participants’ confidence and adherence. This underscores the importance of integrating exercise professionals into mental health settings (Furzer et al., 2021) especially for those who may be deconditioned (McMahen et al., 2024). Interestingly, DOMS is rarely reported in prior RT studies in this population, potentially due to lighter intensity protocols (Maurus et al., 2020; Şenormancı et al., 2021) or a lack of reporting (Heggelund et al., 2012; Silva et al., 2015). This study’s high adherence underscores the importance of proactively addressing exercise-related barriers.

Several improvements were found in the RT group but not the AIT group. Significant strength improvements were observed, consistent with expectations for untrained individuals engaging in regular RT and extend several other RT studies in people with psychotic disorders (Gallardo-Gómez et al., 2023; Heggelund et al., 2012; Silva et al., 2015). People with schizophrenia typically have lower bone mineral density and higher rates of osteoporosis than healthy controls (Stubbs et al., 2014), particularly those over 40 years of age, and/or who may be taking prolactin rising antipsychotics (Tseng et al., 2015). RT may have a specific application to address bone health and mitigate against the increased risk of fractures and falls in this population (Kishimoto et al., 2012). Future studies should further investigate RT’s impact on bone mineral density in people with psychotic disorders.

The RT group engaged in more self-reported total weekly PA post-intervention compared to the AIT group, which may be due to improved muscular conditioning in RT participants, encouraging additional incidental PA. Conversely, AIT participants might have reduced incidental PA outside structured sessions, perceiving they had already met aerobic exercise requirements. Further qualitative analysis of RT participants’ experiences could provide deeper insights into this outcome and consideration of future objective PA monitoring to reduce any influence of recall bias (Soundy et al., 2014).

The RT group also showed within-group improvements in psychiatric and negative symptoms. While modest, the negative symptom improvements met clinically significant thresholds after just 8 weeks (Leucht et al., 2019), aligning with findings from two moderate-intensity RT studies (García-Garcés et al., 2021b; Silva et al., 2015).

No significant changes in global or physical functioning were observed between exercise groups, contrasting with prior studies that reported improvements, predominantly from aerobic exercise (Korman et al., 2023). The brief duration of the intervention (60 days) may have been insufficient to detect changes, especially given the WHODAS assesses functioning over the past 30 days. Supporting individuals with psychotic disorders to perform daily tasks independently remains critical for functional recovery, as they often perform such tasks at levels akin to individuals three decades older (Harvey and Strassnig, 2012).

Contrary to expectations, being randomly assigned to a preferred exercise type did not affect participation rates. The psychological need for autonomy has been considered important for exercise engagement (Teixeira et al., 2012) and relevant for PA adoption in people with schizophrenia (Firth et al., 2016a; Vancampfort et al., 2015b). Furthermore, interventions that allowed for individual exercise preference have been associated with higher adherence in people with schizophrenia (Kimhy et al., 2015). However, a third of the sample had no exercise preference at baseline, which may explain the lack of association between preference and participation. Further, access to skilled exercise professionals may also have made exercise more acceptable and engaging (Vancampfort et al., 2016) regardless of randomisation.

### Strengths

This intervention was delivered by exercise professionals, aligning with the preferences of people with SMI. This intervention was designed using the COM-B behaviour change model and interventions designed using behaviour change models have been associated with an increase in PA (Romain et al., 2020). Furthermore, the intervention was developed based on prior implementation experiences and included strategies such as integration of exercise within the multidisciplinary team, prioritisation of exercise by mental health staff (Lederman et al., 2017), support of participants for attendance and reinforcement of gains (Arnautovska et al., 2022). These factors likely contributed to the successful implementation of the overall intervention.

### Limitations

This pragmatic RCT was not powered to detect differences in secondary outcomes between two active exercise conditions. A larger, adequately powered trial of longer duration is recommended to examine differences between RT and other exercise types on psychological and global functioning, as well as their comparative effectiveness alongside other mental health interventions, such as cognitive remediation and cognitive-behavioural therapy (Young et al., 2022). While isolating RT enabled a focused evaluation of its implementation and effectiveness, general population recommendations emphasise combining aerobic and RT for optimal health benefits (Bull et al., 2020) and future research should also explore this combination with mental health interventions. The absence of an inactive control group was due to practical challenges in assigning participants sharing communal living or gym spaces to a control condition and the risk of contamination (Robinson et al., 2020). Future studies could address this limitation through a cluster-controlled design involving multiple sites. The study was a pragmatic trial (Patsopoulos, 2011), offering important information about RT within a real-world setting and may be relevant to other rehabilitation or community settings where improving holistic health outcomes are a strong focus; however, findings may not be generalisable to more acute psychiatric settings.

The study faced implementation challenges, including staffing inconsistencies (Czosnek et al., 2023) exacerbated by COVID 19, and many adjustments were required to adapt to threats to consistent resourcing of the intervention. Having permanent exercise professional integrated within multidisciplinary teams (Fibbins et al., 2019) and leadership to prioritise exercise as an ongoing core intervention within mental health service models are important strategies to address these challenges in future (Rosenbaum et al., 2018).

## Conclusion

RT was feasible and acceptable to people living with psychotic disorders, with no serious AEs and comparable to AIT. RT resulted in a higher amount of post-intervention self-reported PA compared to AIT. Further research is warranted to explore this further. RT was associated with strength improvements across serval muscle groups, and psychiatric symptoms, but not global or other aspects of physical functioning. A larger trial over a longer period is required to evaluate changes in both psychiatric symptoms and global functioning in RT compared to aerobic exercise and in comparison with other psychosocial therapies. Impact of preference for exercise type on outcomes needs further investigation.

## Supplemental Material

sj-docx-1-anp-10.1177_00048674251361681 – Supplemental material for The feasibility of resistance training versus aerobic exercise in a rehabilitation setting for people living with psychotic disorders: A randomised controlled trial Supplemental material, sj-docx-1-anp-10.1177_00048674251361681 for The feasibility of resistance training versus aerobic exercise in a rehabilitation setting for people living with psychotic disorders: A randomised controlled trial by Nicole Korman, Robert Stanton, Mike Trott, Brendon Stubbs, Andrea Baker, Cassandra Butler, Dan Siskind, Simon Rosenbaum, Joseph Firth, Rebecca Martland, Talia McIntosh, Nicola Warren, Edward Heffernan, Frances Dark and Justin Chapman in Australian & New Zealand Journal of Psychiatry

sj-docx-2-anp-10.1177_00048674251361681 – Supplemental material for The feasibility of resistance training versus aerobic exercise in a rehabilitation setting for people living with psychotic disorders: A randomised controlled trial Supplemental material, sj-docx-2-anp-10.1177_00048674251361681 for The feasibility of resistance training versus aerobic exercise in a rehabilitation setting for people living with psychotic disorders: A randomised controlled trial by Nicole Korman, Robert Stanton, Mike Trott, Brendon Stubbs, Andrea Baker, Cassandra Butler, Dan Siskind, Simon Rosenbaum, Joseph Firth, Rebecca Martland, Talia McIntosh, Nicola Warren, Edward Heffernan, Frances Dark and Justin Chapman in Australian & New Zealand Journal of Psychiatry

sj-docx-3-anp-10.1177_00048674251361681 – Supplemental material for The feasibility of resistance training versus aerobic exercise in a rehabilitation setting for people living with psychotic disorders: A randomised controlled trial Supplemental material, sj-docx-3-anp-10.1177_00048674251361681 for The feasibility of resistance training versus aerobic exercise in a rehabilitation setting for people living with psychotic disorders: A randomised controlled trial by Nicole Korman, Robert Stanton, Mike Trott, Brendon Stubbs, Andrea Baker, Cassandra Butler, Dan Siskind, Simon Rosenbaum, Joseph Firth, Rebecca Martland, Talia McIntosh, Nicola Warren, Edward Heffernan, Frances Dark and Justin Chapman in Australian & New Zealand Journal of Psychiatry

sj-docx-4-anp-10.1177_00048674251361681 – Supplemental material for The feasibility of resistance training versus aerobic exercise in a rehabilitation setting for people living with psychotic disorders: A randomised controlled trial Supplemental material, sj-docx-4-anp-10.1177_00048674251361681 for The feasibility of resistance training versus aerobic exercise in a rehabilitation setting for people living with psychotic disorders: A randomised controlled trial by Nicole Korman, Robert Stanton, Mike Trott, Brendon Stubbs, Andrea Baker, Cassandra Butler, Dan Siskind, Simon Rosenbaum, Joseph Firth, Rebecca Martland, Talia McIntosh, Nicola Warren, Edward Heffernan, Frances Dark and Justin Chapman in Australian & New Zealand Journal of Psychiatry

sj-docx-5-anp-10.1177_00048674251361681 – Supplemental material for The feasibility of resistance training versus aerobic exercise in a rehabilitation setting for people living with psychotic disorders: A randomised controlled trial Supplemental material, sj-docx-5-anp-10.1177_00048674251361681 for The feasibility of resistance training versus aerobic exercise in a rehabilitation setting for people living with psychotic disorders: A randomised controlled trial by Nicole Korman, Robert Stanton, Mike Trott, Brendon Stubbs, Andrea Baker, Cassandra Butler, Dan Siskind, Simon Rosenbaum, Joseph Firth, Rebecca Martland, Talia McIntosh, Nicola Warren, Edward Heffernan, Frances Dark and Justin Chapman in Australian & New Zealand Journal of Psychiatry

sj-docx-6-anp-10.1177_00048674251361681 – Supplemental material for The feasibility of resistance training versus aerobic exercise in a rehabilitation setting for people living with psychotic disorders: A randomised controlled trial Supplemental material, sj-docx-6-anp-10.1177_00048674251361681 for The feasibility of resistance training versus aerobic exercise in a rehabilitation setting for people living with psychotic disorders: A randomised controlled trial by Nicole Korman, Robert Stanton, Mike Trott, Brendon Stubbs, Andrea Baker, Cassandra Butler, Dan Siskind, Simon Rosenbaum, Joseph Firth, Rebecca Martland, Talia McIntosh, Nicola Warren, Edward Heffernan, Frances Dark and Justin Chapman in Australian & New Zealand Journal of Psychiatry

sj-docx-7-anp-10.1177_00048674251361681 – Supplemental material for The feasibility of resistance training versus aerobic exercise in a rehabilitation setting for people living with psychotic disorders: A randomised controlled trial Supplemental material, sj-docx-7-anp-10.1177_00048674251361681 for The feasibility of resistance training versus aerobic exercise in a rehabilitation setting for people living with psychotic disorders: A randomised controlled trial by Nicole Korman, Robert Stanton, Mike Trott, Brendon Stubbs, Andrea Baker, Cassandra Butler, Dan Siskind, Simon Rosenbaum, Joseph Firth, Rebecca Martland, Talia McIntosh, Nicola Warren, Edward Heffernan, Frances Dark and Justin Chapman in Australian & New Zealand Journal of Psychiatry

sj-docx-8-anp-10.1177_00048674251361681 – Supplemental material for The feasibility of resistance training versus aerobic exercise in a rehabilitation setting for people living with psychotic disorders: A randomised controlled trial Supplemental material, sj-docx-8-anp-10.1177_00048674251361681 for The feasibility of resistance training versus aerobic exercise in a rehabilitation setting for people living with psychotic disorders: A randomised controlled trial by Nicole Korman, Robert Stanton, Mike Trott, Brendon Stubbs, Andrea Baker, Cassandra Butler, Dan Siskind, Simon Rosenbaum, Joseph Firth, Rebecca Martland, Talia McIntosh, Nicola Warren, Edward Heffernan, Frances Dark and Justin Chapman in Australian & New Zealand Journal of Psychiatry
