# Supplementary material for: The feasibility of resistance training versus aerobic exercise in a rehabilitation setting for people living with psychotic disorders: A randomised controlled trial
Source: Aust N Z J Psychiatry. 2025 Nov 11;60(6):538–52. doi: 10.1177/00048674251361681 (PMC13191080; doi:10.1177/00048674251361681)
Supplement: sj-docx-1-anp-10.1177_00048674251361681 – Supplemental material for The feasibility of resistance training versus aerobic exercise in a rehabilitation setting for people living with psychotic disorders: A randomised controlled trial [file sj-docx-1-anp-10.1177_00048674251361681.docx]

**Appendix 1.** Schedule of visits and assessments

| ***Visit*** | 0  (Screening  Phase) | 1  (Baseline) | 2 | 3 | 4 | 5 | 6 | 7 | 8 | 9 |
| --- | --- | --- | --- | --- | --- | --- | --- | --- | --- | --- |
| ***Week*** |  | 0 | 1 | 2 | 3 | 4 | 5 | 6 | 7 | 8 |
| **SCREENING AND CONSENT** | | | | | | | | | |  |
| Informed consent | x |  |  |  |  |  |  |  |  |  |
| Ongoing capacity | x |  |  |  |  |  |  |  |  |  |
| Inclusion / exclusion criteria | x |  |  |  |  |  |  |  |  |  |
| Baseline demographics | x |  |  |  |  |  |  |  |  |  |
| Physical Health measures (BP, height, weight, waist circumference) | x |  |  |  |  |  |  |  |  |  |
| **SAFETY** | | | | | | | | | |  |
| Adverse events (before/after each session by the AEP) |  |  | x | x | x | x | x | x | x | x |
| **EFFECTIVENESS** | | | | | | | | | |  |
| Accelerometry (worn for 5 days prior to randomisation) |  | x |  |  |  |  |  |  |  |  |
| SIMPAQ |  | x |  |  |  |  |  |  |  |  |
| Preferences, Barriers, Motivators Questionnaire |  | x |  |  |  |  |  |  |  |  |
| WHODAS 2.0 |  | x |  |  |  |  |  |  |  | x |
| BPRS |  | x |  |  |  |  |  |  |  | x |
| SANS |  | x |  |  |  |  |  |  |  | x |
| Physical Capacity (6MWT, 30 sec sit-to-stand, grip strength, push up test) |  | x |  |  |  |  |  |  |  | x |
| **INTERVENTION SESSIONS** | | | | | | | | | | |
| RT and AIT (x3 sessions per week) |  |  | x | x | x | x | x | x | x | x |
| Health Coaching Sessions offered |  |  | x |  | x |  | x |  | x |  |
| **ACCEPTABILITY** | | | | | | | | | | |
| Acceptability questionnaire |  |  |  |  |  |  |  |  |  | x |
|  |  |  |  |  |  |  |  |  |  |  |
| Individual interview (RT condition) - optional |  |  |  |  |  |  |  |  |  | x |

Abbreviations: AIT – aerobic interval training, RT – resistance training, SIMPAQ - Simple Physical Activity Questionnaire, WHODAS 2.0 – World Health Organisation Disability Adjusted Scale version 2, SANS – Scale Assessment of Negative Symptoms, BPRS – Brief Psychiatric Rating Scale, 6MWT - Six-minute walk test, BP – Blood pressure, AEP – accredited exercise physiologist.
