# Supplementary material for: The feasibility of resistance training versus aerobic exercise in a rehabilitation setting for people living with psychotic disorders: A randomised controlled trial
Source: Aust N Z J Psychiatry. 2025 Nov 11;60(6):538–52. doi: 10.1177/00048674251361681 (PMC13191080; doi:10.1177/00048674251361681)
Supplement: sj-docx-3-anp-10.1177_00048674251361681 – Supplemental material for The feasibility of resistance training versus aerobic exercise in a rehabilitation setting for people living with psychotic disorders: A randomised controlled trial [file sj-docx-3-anp-10.1177_00048674251361681.docx]

**Appendix 3.** Proforma CERT checklist

| **Section** | **Item number** | **Description** | **Details** | **Location (page)** |
| --- | --- | --- | --- | --- |
| WHAT:  materials | 1 | Detailed description of the type of exercise equipment | RT - hand weights, kettle bells, resistance bands and a chest press bar, squat rack and floor mat appropriate for small clinical gym settings to enable replication of the study without substantial investment in specialised equipment or gym spaces. AIT - equipment included a stationary bike, treadmill, rowing ergometer and elliptical machine. | Materials and Methods: Resistance Training Intervention and Aerobic Interval Training, page 7-8 |
| WHO:  provider | 2 | Detailed description of the qualifications, teaching/supervising expertise, and/or training  undertaken by the exercise instructor | Fully supervised by an accredited exercise physiologist (AEP), with experience in mental health settings, and in delivering exercise to untrained and vulnerable health populations  Training for AEP’s was conducted in the specific delivery of the intervention such that participant programs were be standardised for each condition | Material and Methods: Interventional Exercise(s), page 5  Material and Methods: Fidelity, page 8 |
| HOW:  delivery | 3 | Describe whether exercises are performed individually or in a group | Participants choose whether to engage in individual sessions or in small groups.  During COVID 19 pandemic all participants were only offered individual sessions. | Material and Methods: Strategies to enhance endurance, page 6  Results: Real world trial considerations, page 14 |
|  | 4 | Describe whether exercises are supervised or unsupervised and how they are delivered | All intervention sessions fully supervised by an accredited exercise physiologist (AEP) | Material and Methods: Interventional Exercise(s), page 5 |
|  | 5 | Detailed description of how adherence to exercise is measured and reported | Participation in an individual session was recorded if greater than 50% of the session was attended. Individual participation in ≥ 65% of the 24 available sessions (16 /24 sessions) was considered feasible. Feasibility was considered if greater than 70% of participants allocated per condition completed the weight week intervention | Material and Methods: Primary outcomes, page 9-10 |
|  | 6 | Detailed description of motivation strategies | 15-minute fortnightly health coaching session focusing on self-monitoring, goal setting, and building self-efficacy for exercise. Participants were encouraged to play music of their choice to enhance pleasure and could choose whether to engage in individual sessions or in small groups. The intervention was based on the capability, opportunity and motivation (COM-B) model of behaviour change (Michie et al., 2011)and theoretical domains of the COM-B model have been articulated by the Theoretical Domains Framework (TDF) used in health care interventions (Atkins et al., 2017). | Material and methods: Strategies to enhance endurance, page 6 |
|  | 7a | Detailed description of the decision rule(s) for determining exercise progression | Both conditions were aimed at moderate intensity exercise that was balanced for total number of sessions and volume. Active exercise duration started as 30-minutes duration. Volume was progressed to 40 minutes per session by week four of the intervention, with total duration of the session approximately 55 minutes, including warm up and cool down.Exercise prescription was reviewed weekly by the AEP and exercise progressed/regressed according to individual response. |  |
|  | 7b | Detailed description of how the exercise program was progressed | Intensity of the intervention was monitored using the Borg Category Ratio 10 scale of Perceived Exertion scale (RPE), (Heath, 1998)- a 10 point scale to assess subjective perception of effort that has been calibrated against exercise intensity and will guide intensity of exercise prescription. The aim was a starting RPE of 2-3 in week 1, with progression to RPE 4 by week 3. |  |
|  | 8 | Detailed description of each exercise to enable replication (e.g. photographs, illustrations ,  video etc) | The content and weights of each session was recorded using standard protocols. | Supplementary material X; example of an RT and and AIT program  Supplementary material X: photos of each session |
|  | 9 | Detailed description of any home program component (e.g. other exercises, stretching etc) | No specific home program was prescribed, however participants could engage in the same program developed for the intervention in their own time, but there were study restrictions around engaging in other exercise outside of the randomised condition to prevent contamination of results. | Materials and Methods:  Randomisation, page 4 |
|  | 10 | Describe whether there are any non-exercise components (e.g. education, cognitive  behavioural therapy, massage etc) | 15-minute fortnightly health coaching session focusing on self-monitoring, goal setting, and building self-efficacy for exercise. | Material and methods: Strategies to enhance endurance, page 6 |
|  | 11 | Describe the type and number of adverse events that occurred during exercise | Adverse events (AE’s) were monitored closely during the intervention using an AE protocol  There were 3 serious adverse events, none related to the exercise intervention with no difference between groups. There were 39 minor adverse events related to exercise; 34 in the RT group versus 5 in the AIT group, with the majority of RT-related AE’s being muscle soreness (n=22) or joint stiffness (n=7). The RT group had a significantly higher number of AE’s per participant compared to AIT, z= 3.23, p=0.01, see Figure 2. There were no drop- outs due to exercise -related AE’s in either group | Material and methods: primary outcomes, Adverse events, page 9  Material and methods: Primary outcomes, page 13 and Table 2a |
| WHERE:  location | 12 | Describe the setting in which the exercises are performed | The study was conducted within the shared courtyard, using pre-existing gym facilities and equipment maintained by each residential rehabilitation unit. | Material and Methods: Interventional Exercise(s), page 5 |
| WHEN, HOW MUCH:  dosage | 13 | Detailed description of the exercise intervention including, but not limited to, number of  exercise repetitions/sets/sessions, session duration, intervention/program duration etc | Sessions involved a warm-up (AIT - 10-minute aerobic warm up; RT - 5-minute aerobic & 5-minute stretching warm up), active intervention and 5-minute cool down phase. Active exercise duration started as 30-minutes duration. Volume was progressed to 40 minutes per session by week four of the intervention, with total duration of the session approximately 55 minutes, including warm up and cool down.  RT - to occur on 2 or more days per week, involving main muscle groups. An initial assessment of muscle strength was assessed using the 12 repetition maximum test (12-RM) for relevant large muscle groups (the squat and bench press) to guide exercise prescription. Participants completed three to four sets of 8-12 repetitions to moderate fatigue (RPE 3-4/10), and approximately 90 seconds passive rest interval between sets. Week 1 started with 40% of 1-RM. As strength increased, the RT was progressed using heavier weights, as per capacity of the individual and in accordance with their RPE. Approximately six to eight exercises across main muscle groups used in activities of daily living were prescribed per session until a total of 30 minutes of active exercise was completed.  The AEP will aim for the participant to maintain an RPE at 2–3 for the first 2 weeks, then increased to a minimum RPE of 4/10 by week 3 and further increased each week as per individual participant's capacity. Following warm-up, participants will begin interval sets, beginning with four minutes of moderate intensity exercise (RPE 3-4) interspersed with three minutes of active recovery (reduced intensity interval, RPE 1- 2) until a total of 30 minutes of AIT is completed. Volume will be progressed to 40 minutes by week 4. | Material and Methods: Interventional Exercise(s), page 5  Material and Methods: Resistance Training, page 6  Material and Methods: Aerobic Interval training, page 7 |
| TAILORING:  What, how | 14a | Describe whether the exercises are generic (one size fits all) or tailored whether tailored to  the individual | Exercise prescription was reviewed weekly by the AEP and exercise progressed/regressed according to individual response. | Material and Methods: Interventional Exercise(s), page 6 |
|  | 14b | Detailed description of how exercises are tailored to the individual | Equipment was chosen as the resistance and speed could be modified to suit individual participant capacity and preference. | Material and Methods: Aerobic Interval training, page 8 |
|  | 15 | Describe the decision rule for determining the starting level at which people commence an  exercise program (such as beginner, intermediate, advanced etc) | The aim was a starting RPE of 2-3 in week 1, with progression to RPE 4 by week 3.  RT - An initial assessment of muscle strength was assessed using the 12 repetition maximum test (12-RM) for relevant large muscle groups (the squat and bench press) to guide exercise prescription.  AIT - Initial prescription was aimed at an intensity achievable for participants with low initial CRF but progressed to reach moderate intensity by week 3 | Material and Methods: Interventional Exercise(s), page 5  Material and Methods: Resistance Training, page 7  Material and Methods: Aerobic Interval Training, page 8 |
| HOW WELL:  planned, actual | 16a | Describe how adherence or fidelity to the exercise intervention is assessed/measured | Programs were reviewed against protocol criteria at intervals by the research team to ensure prescriptions were conducted within the scope of the intervention. | Material and Methods: Fidelity, page 8 |
|  | 16b | Describe the extent to which the intervention was delivered as planned | Completion of the entire eight week intervention rates were 24/27 (88.8%) in each condition, with no difference between the groups. Median participation rates were 23.5 (IQR 4) out of a total of 24 sessions, and no difference between groups. | Results: Primary outcomes, page 13 |

Abbreviations: RT – resistance training, AIT – aerobic interval training

**References**

Atkins, L., Francis, J., Islam, R., O'Connor, D., Patey, A., Ivers, N., Foy, R., Duncan, E. M., Colquhoun, H., Grimshaw, J. M., Lawton, R., & Michie, S., 2017. A guide to using the Theoretical Domains Framework of behaviour change to investigate implementation problems. Implement Sci, 12(1), 77-77. doi:10.1186/s13012-017-0605-9

Heath, E. M., 1998. Borg's Perceived Exertion and Pain Scales. Medicine& Science in Sports & Exercise, 30(9), 1461. doi:10.1249/00005768-199809000-00018

Michie, S., van Stralen, M. M., & West, R., 2011. The behaviour change wheel: A new method for characterising and designing behaviour change interventions.(Research). Implementation Science, 6, 42.
