# Supplementary material for: The feasibility of resistance training versus aerobic exercise in a rehabilitation setting for people living with psychotic disorders: A randomised controlled trial
Source: Aust N Z J Psychiatry. 2025 Nov 11;60(6):538–52. doi: 10.1177/00048674251361681 (PMC13191080; doi:10.1177/00048674251361681)
Supplement: sj-docx-4-anp-10.1177_00048674251361681 – Supplemental material for The feasibility of resistance training versus aerobic exercise in a rehabilitation setting for people living with psychotic disorders: A randomised controlled trial [file sj-docx-4-anp-10.1177_00048674251361681.docx]

**Appendix 4.** Example of a typical Resistance Training (RT) prescription

| **EXERCISE** | **IMAGE** |
| --- | --- |
| Chest press | 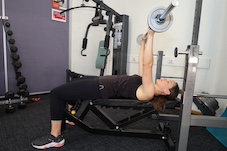 |
| Goblet Squat | 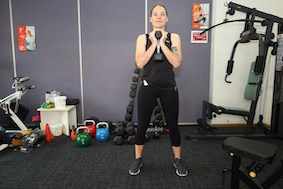  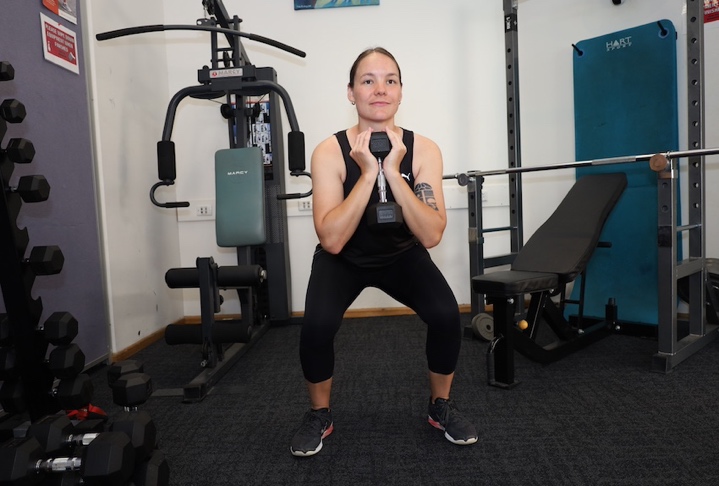 |
| Kettle bell core twist | 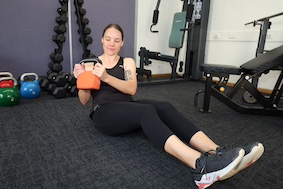 |
| Medicine ball slam | 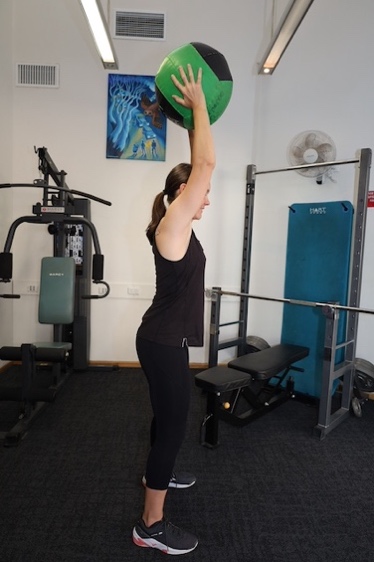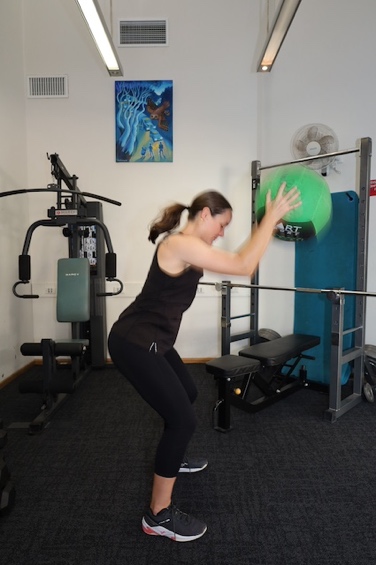 |
| Lat pull down | 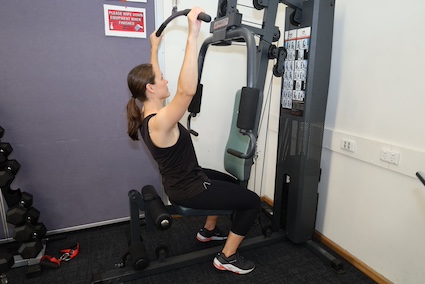 |
| Dead bug | 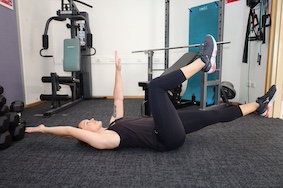 |
| Squat | 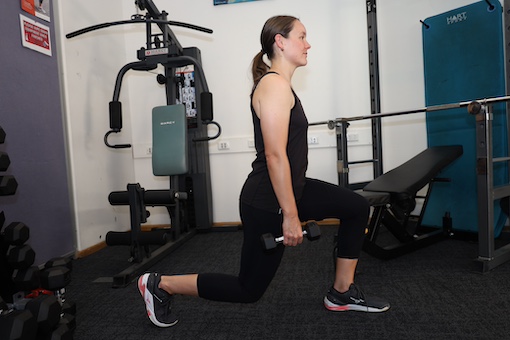 |
| Romanian kettle bell | 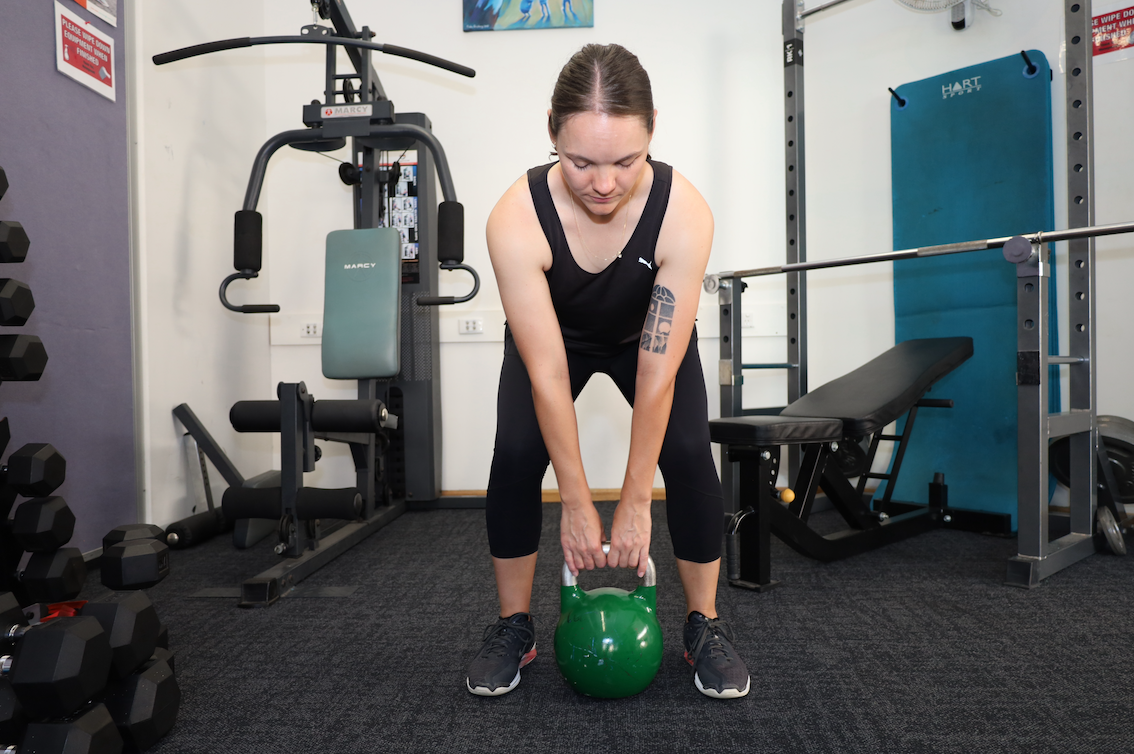 |
