# Supplementary material for: The feasibility of resistance training versus aerobic exercise in a rehabilitation setting for people living with psychotic disorders: A randomised controlled trial
Source: Aust N Z J Psychiatry. 2025 Nov 11;60(6):538–52. doi: 10.1177/00048674251361681 (PMC13191080; doi:10.1177/00048674251361681)
Supplement: sj-docx-5-anp-10.1177_00048674251361681 – Supplemental material for The feasibility of resistance training versus aerobic exercise in a rehabilitation setting for people living with psychotic disorders: A randomised controlled trial [file sj-docx-5-anp-10.1177_00048674251361681.docx]

**Appendix 5.** COM-B and TDF domains with intervention techniques.

| **COM-B Domain** | **TDF Domain** | **Definition** | **Intervention technique** |
| --- | --- | --- | --- |
| **Capability** | KNOWLEDGE | *An awareness of the existence of something* | AEP imparted knowledge at the start of the intervention about the exercise type, together with expected benefits through 1:1 discussion with the participant |
|  | SKILLS | *An ability or proficiency acquired through practice* | RT and aerobic exercise skill development was developed through instruction, demonstration and repetitive practice with the AEP in supervised sessions throughout the intervention |
|  | BEHAVIOURAL REGULATION | *Anything aimed at managing or changing objectively observed or measured actions*. | -Participants were encouraged to attend 3*/week for best outcomes  -Schedule clashes were highlighted and assistance to resolve them between the participant, AEP and the MDT staff to promote highest adherence. |
|  | MEMORY, ATTENTION AND DECISION PROCESSES | *The ability to retain information, focus selectively on aspects of the environment and choose between two alternatives* | -AEP checked in on recall of information about exercise knowledge, and skills at intervals throughout the intervention.  -use of rehabilitation calendar to prompt recall of session date and time |
| **Opportunity** | ENVIRONMENTAL CONTEXT AND RESOURCES | *Any circumstances of a person's situation that encourages or discourages the development of skills and abilities, independence, social competence and adaptive behaviour* | -Provision of fully supervised RT or AIT session, 3*/week. Led by a qualified AEP.  -Gym provided on site to participant with necessary equipment. Orientation to gym equipment provided at the start of the intervention to encourage confidence and independent use.  -MDT staff entered exercise appointments into the electronic calendar of the individual which were then provided each week to the participant.  -Participants were prompted to check their calendar, and reminded of their appointments on the morning of the intervention and  -supported to consider planning for appropriate dress and attendance at the gym, including breakfast and hydration. |
|  | SOCIAL INFLUENCES | *Interpersonal processes that can cause individuals to change their thoughts, feelings or behaviours* | -Encouragement and emotional support to attend and participate provided by MDT staff, the AEP and via health coaching sessions.  -Residents of the facility encouraged each other to seek participation in the study via peer influences. |
| **Motivation** | SOCIAL/PROFESSIONAL ROLE AND IDENTITY | *A coherent set of behaviours and displayed personal qualities of an individual in a social or work setting* | -Participants were encouraged to see their participation as developing an identity as someone who chooses healthy behaviours in health coaching sessions |
|  | BELIEFS ABOUT CAPABILITIES | *Acceptance of the truth, reality or validity about talent, ability or facility that a person can put to constructive use* | Participants were encouraged at each session and the end of the intervention to reflect on the development of their skills and to consider future capabilities to consider exercising beyond the intervention |
|  | BELIEFS ABOUT CONSEQUENCES | *Acceptance of the truth, reality or validity about outcomes of a behaviour in a given situation* | Participants were provided with feedback about improvements in their health across various parameters at the end of the intervention (ie changes in strength, fitness or functional fitness, mental health) |
|  | GOALS | *Mental representations of outcomes or end states that an individual wants to achieve* | Personalised goals were elicited in the first session and goal achievement was revisited by the AEP each session and at the end of the intervention. |
|  | EMOTION | *A complex reaction pattern, involving experiential, behavioural and psychological elements* | Participants were encouraged to consider psychological and emotional response to exercise sessions which was also measured at week 3 and week 8. |
|  | OPTIMISM | *The confidence that things will happen for the best or a desired goal with be obtained* | Encouraged during friendly, positive interactions about the possible consequences of participation both with the AEP and MDT staff supporting to attend. |
|  | INTENTIONS | *A conscious decision to perform a behaviour* |  |
|  | REINFORCEMENT | *Increasing the probability of a response by arranging a dependent relationship, or contingency between response and stimuli* | Specific positive feedback about attendance and participation at each session was provided by the AEP in close proximity to the exercise session, repeatedly and often to provide reinforcement.  Small incentives provided for adherence – a water bottle for full attendance week 3. |

Abbreviations: AEP – Accredited Exercise Physiologist, RT – resistance training, AIT – aerobic training, COM-B – Capability, opportunity, motivation. TDF – Theoretical Domain Framework
