# Supplementary material for: The feasibility of resistance training versus aerobic exercise in a rehabilitation setting for people living with psychotic disorders: A randomised controlled trial
Source: Aust N Z J Psychiatry. 2025 Nov 11;60(6):538–52. doi: 10.1177/00048674251361681 (PMC13191080; doi:10.1177/00048674251361681)
Supplement: sj-docx-6-anp-10.1177_00048674251361681 – Supplemental material for The feasibility of resistance training versus aerobic exercise in a rehabilitation setting for people living with psychotic disorders: A randomised controlled trial [file sj-docx-6-anp-10.1177_00048674251361681.docx]

**Appendix 6: Exercise Acceptability questionnaire**

**How strongly do you agree or disagree with the following statements about your exercise program?**

|  |  | Strongly Disagree | Disagree | Neither agree nor disagree | Agree | Strongly Agree |
| --- | --- | --- | --- | --- | --- | --- |
| a | I enjoyed the exercise program | 🞎 | 🞎 | 🞎 | 🞎 | 🞎 |
| b | I found the exercise program was easy too difficult for me | 🞎 | 🞎 | 🞎 | 🞎 | 🞎 |
| c | I found the exercise program helped me to be able to carry out daily tasks more easily (ie shopping, cleaning) | 🞎 | 🞎 | 🞎 | 🞎 | 🞎 |
| d | The exercise program was fun to do | 🞎 | 🞎 | 🞎 | 🞎 | 🞎 |
| e | I wanted to stop this type of exercise before the study ended | 🞎 | 🞎 | 🞎 | 🞎 | 🞎 |
| f | I found the exercise program was challenging enough for me | 🞎 | 🞎 | 🞎 | 🞎 | 🞎 |
| g | I will want to keep this type of exercise going after the study finishes | 🞎 | 🞎 | 🞎 | 🞎 | 🞎 |
| h | I looked forward to these exercise sessions with my trainer | 🞎 | 🞎 | 🞎 | 🞎 | 🞎 |
| i | Three of these exercise sessions per week was too much for me | 🞎 | 🞎 | 🞎 | 🞎 | 🞎 |
| j | The exercise program helped my mental health improve | 🞎 | 🞎 | 🞎 | 🞎 | 🞎 |
| K | I wished I could have changed to a different sort of exercise than the one I am doing during the study | 🞎 | 🞎 | 🞎 | 🞎 | 🞎 |
| l | I like changes I have noticed in my body after completing this exercise program | 🞎 | 🞎 | 🞎 | 🞎 | 🞎 |
| m | I would feel confident to continue this sort of exercise on my own after the study finishes | 🞎 | 🞎 | 🞎 | 🞎 | 🞎 |
| n | Individual sessions of this type of exercise made my mood improve | 🞎 | 🞎 | 🞎 | 🞎 | 🞎 |

1. Please comment on any other features of the exercise program you found useful?
2. Is there anything else about the exercise program that was unpleasant or difficult – please explain?
3. Any other feedback about the use of exercise program in this study you would like to provide?
