# Supplementary material for: The feasibility of resistance training versus aerobic exercise in a rehabilitation setting for people living with psychotic disorders: A randomised controlled trial
Source: Aust N Z J Psychiatry. 2025 Nov 11;60(6):538–52. doi: 10.1177/00048674251361681 (PMC13191080; doi:10.1177/00048674251361681)
Supplement: sj-docx-7-anp-10.1177_00048674251361681 – Supplemental material for The feasibility of resistance training versus aerobic exercise in a rehabilitation setting for people living with psychotic disorders: A randomised controlled trial [file sj-docx-7-anp-10.1177_00048674251361681.docx]

**Appendix 7a.** Preferences, enablers and barriers - Baseline questionnaire.

| ***PREFERENCES QUESTIONNAIRE*** | **Yes** | **No** | **Unsure/no preference** |
| --- | --- | --- | --- |
| Have you ever done RT before? | 47 (87%) | 7 (13%) |  |
| Have you done RT in the last 3 months? | 36 (67%) | 11 (20.4%) |  |
| Have you ever done AIT before? | 37 (68.5%) | 17 (31.5%) |  |
| Have you done AIT in the last 3 months? | 23 (42.6%) | 14 (26%) |  |
| For this intervention – do you have a preference for RT, AIT, or no preference? | RT – 13 (24.5%) | AIT – 22 (41.5%) | No preference – 18 (34%) |
| **Do you have a preference for the following exercise types:** | | | |
| -jogging | 24 (44.4%) | 30 (55.6%) |  |
| -gym based cardio | 44 (81.5%) | 10 (18.5%) |  |
| -cycling | 34 (63%) | 20 (37%) |  |
| -swimming | 23 (42.6%) | 31 (57.4%) |  |
| -gym based weights | 44 (81.5%) | 10 (18.5%) |  |
| -fitness classes | 37 (68.5%) | 17 (31.5%) |  |
| -tennis | 27 (50%) | 27 (50%) |  |
| -soccer/ rugby/ football | 27 (54%) | 27 (50%) |  |
| -walking | 52 (96.3%) | 2 (3.7%) |  |
| -boxing | 28 (51.9%) | 26 (48.9%) |  |
| -dancing | 15 (27.8%) | 39 (72.2%) |  |
| **Do you have a preference for the following setting:** | | | |
| -group PA | 42 (77.8%) | 12 (22,2%) |  |
| -exercise alone | 42 (77.8%) | 12 (22,2%) |  |
| -private gym | 33 (61.1%) | 21 (38.9%) |  |
| -CCU facilities | 53 (98.1%) | 1 (1.9%) |  |
| -home exercise | 36 (66.7%) | 18 (33.3%) |  |

Abbreviations: RT – resistance training, AIT – aerobic interval training, PA – physical activity.

**Appendix 7b.** Attitudes and perceived barriers to physical activity

| ***ATTITUDE ITEMS*** | **Strongly disagree** | **Disagree** | **neutral** | **Agree** | **Strongly agree** |
| --- | --- | --- | --- | --- | --- |
| **I engage in physical activity:** | | | | | |
| -in order to improve my fitness |  | 4(7.4%) | 5 (9.3%) | 26 (48.1%) | 19 (35.2%) |
| -to lose weight | 2 (3.7%0 | 8(14.8%) | 9(16.7%) | 23(42.6%) | 12 (22.2%) |
| -to improve strength | 1(1.9%0 | 6(11.1%) | 6(11.1%) | 31(57.4%) | 10 (18.5%) |
| -to improve my appearance | 2(3.7%) | 8(14.8%) | 10(18.5%) | 23(42.6%) | 11(20.4%) |
| -to improve sleep quality | 2(3.7%) | 12(22.2%) | 11(20.4%) | 21(38.9%) | 8(14.8%) |
| -because my doctor advised me to (or other HP) | 6 (11.1%) | 12(22.2%) | 8(14.8%) | 23(42.6%) | 5(9.3%) |
| -important for my physical health |  | 2(3.7%) | 3(5.6%) | 31(57.4%) | 18(33.3%) |
| -to improve symptoms of my mental illness (other than mood) | 1 (1.9%) | 4(7.4%) | 12(22.2%) | 30(55.6%) | 7(13%) |
| -reduce stress feelings | 1 (3.7%) | 4(7.4%) | 12(22.2%) | 30(55.6%) | 7(13%) |
| -for enjoyment | 2 (3.7%) | 4(7.4%) | 10(18.5%) | 28(51.9%) | 10(18.5%) |
| -to socialise with others | 3 (5.6%) | 11(20.4%) | 13(24.1%) | 23(42.6%) | 4(7.4%) |
| -to improve my mood |  | 1(1.9%) | 14 (25.9%) | 30(55.6%) | 9(16.7%) |
| -when I have more professional support to do so (exercise professional) | 1 (1.9%) | 1 (1.9%) | 6 (11.1%) | 31(57.4%) | 15(27.8%) |
| -for fun/pleasure | 1(1.9%) | 6(11.1%) | 10(18.5%) | 29(53.7%) | 8(14.8%) |
| **The following things stop me from wanting to engage in regular PA** | | | | | |
| -tiredness | 2 (3.7%) | 7 (13%) | 12 (22%) | 28 (51.9%) | 5 (9.3%) |
| -symptoms of my mental illness (other than mood) | 6 (11%) | 20 (37%) | 15 (27.8%) | 13 (24.1%) |  |
| -depressed mood | 5 (9.3%) | 13 (24.1%) | 11 (20.4%) | 19 (35.2%) | 6 (11.1%) |
| -medication side effects | 6 (11.1%) | 20 (37%) | 15 (27.8%) | 13 (24.1%) |  |
| -lack of motivation | 2 (3.7%) | 3 (5.6%) | 8 (14.8%) | 37 (68.5%) | 4 (7.4%) |
| -lack of confidence | 3 (5.6%0 | 18 (33.3%) | 17 (31.5%) | 14 (25.9%) | 2 (3.7%) |
| -Not enough money | 8 (14.8%) | 36 (66.7%) | 4 (7.4%) | 6 (11.1%) |  |
| -unable to access facilities | 8 (14.8%) | 36 (66.7 %) | 4 (7.4%) | 6 (11.1%) |  |
| -don’t have enough support from others | 8 (14.8%) | 34 (63%) | 7 (13%) | 5 (9.3%) |  |
