# Supplementary material for: The feasibility of resistance training versus aerobic exercise in a rehabilitation setting for people living with psychotic disorders: A randomised controlled trial
Source: Aust N Z J Psychiatry. 2025 Nov 11;60(6):538–52. doi: 10.1177/00048674251361681 (PMC13191080; doi:10.1177/00048674251361681)
Supplement: sj-docx-8-anp-10.1177_00048674251361681 – Supplemental material for The feasibility of resistance training versus aerobic exercise in a rehabilitation setting for people living with psychotic disorders: A randomised controlled trial [file sj-docx-8-anp-10.1177_00048674251361681.docx]

**Appendix 8a.** Acceptability Questionnaire Raw Data

|  | *RESISTANCE TRAINING, N=26* | | | | | *AEROBIC INTERVAL TRAINING, N=25* | | | | |
| --- | --- | --- | --- | --- | --- | --- | --- | --- | --- | --- |
|  | N (%) | N (%) | N (%) | N (%) | N (%) | N (%) | N (%) | N (%) | N (%) | N (%) |
| Item number | **Strongly disagree** | **Disagree** | **Neither agree/nor disagree** | **Agree** | **Strongly agree** | **Strongly disagree** | **Disagree** | **Neither agree/nor disagree** | **Agree** | **Strongly agree** |
| Enjoyed the program |  |  | 1 (3.8) | 16 (61.5) | 9 (34.6) |  | 2 (7.7) |  | 9 (34.6) | 14 (53.8) |
| Too difficult | 4 (15.4) | 12(46.2) | 6 (23.1) | 4 (15.4) |  | 4 (15.4) | 6 (23.1) | 11 (42.3) | 4 (15.4) |  |
| Helped with daily tasks |  | 2 (7.7) | 6 (23.1) | 13 (50) | 5 (19.2) |  | 1 (3.8) | 6 (23.1) | 15 (57.7) | 3 (11.5) |
| Program was fun |  |  | 6 (23.1) | 12 (46.2) | 8 (30.8) | 1 (3.8) | 2 (7.7) | 1 (3.8) | 14 (53.8) | 7 (26.9) |
| Wanted to stop | 6 (23.1) | 12 (46.2) | 3 (11.5) | 3 (11.5) |  | 6 (23.1) | 14 (53.8) | 2 (7.7) | 3 (11.5) |  |
| Was challenging enough |  | 5 (19.2) | 1 (3.8) | 17 (65.4) | 3 (11.5) |  | 2 (7.7) | 1 (3.8) | 18 (69.2) | 4 (15.4) |
| Keep this exercise going after the program ends |  | 1 (3.8) | 3 (11.5) | 17 (65.4) | 5 (19.2) | 1 (3.8) | 1 (3.8) | 5 (19.2) | 12 (46.2) | 6 (23.1) |
| Looked forward to the exercise program |  | 2 (7.7) | 4 (15.4) | 15 (57.7) | 5 (19.2) |  | 2 (7.7) | 1 (3.8) | 14 (53.8) | 8 (30.8) |
| Three sessions per week too much | 7 (26.9) | 13 (50) | 4 (15.4) | 2 (7.7) |  | 4 (15.4) | 18 (69.2) | 2 (7.7) | 1 (3.8) |  |
| Exercise helped my mental health |  |  | 4 (15.4) | 18 (69.2) | 4 (15.4) |  |  | 2 (7.7) | 14 (53.8) | 9 (34.6) |
| Wish I could have changed to a different type of exercise | 5 (19.2) | 12 (46.2) | 7 (26.9) | 2 (7.7) |  | 3 (11.5) | 12 (46.2) | 5 (19.2) | 4 (15.4) | 1 (3.8) |
| The program made positive changes to my body |  | 1 (3.8) | 6 (23.1) | 19 (73.1) |  |  | 1 (3.8) | 5 (19.2) | 14 (53.8) | 5 (19.2) |
| Confident to do this on my own after it ends | 1 (3.8) | 1 (3.8) | 4 (15.4) | 16 (16.5) | 4 (15.4) | 1 (3.8) | 2 (7.7) | 3(11.5) | 15 (57.7) | 4 (15.4) |
| Individual sessions improved my mood |  |  | 6 (23.1) | 12 (46.2) | 8 (30.8) |  |  | 1 (3.8) | 16 (61.5) | 8 (30.8) |

**Appendix 8b.** Acceptability Questionnaire – Descriptive analysis and statistical testing.

| **Item number** | **Total sample, agree/strongly agree, n=51** | **Resistance Training, agree/strongly agree, n=26** | **Aerobic Interval Training, agree/strongly agree, n=25** | **Fishers exact:**  **comparison between AIT and RT** |
| --- | --- | --- | --- | --- |
| Enjoyed the program | 48, 94.1% | 25, 96.2% | 23, 92% | 0.61 |
| Too difficult | 8, 15.7% | 4, 15.4% | 4, 16% | 1.00 |
| Helped with daily tasks | 36, 76.2% | 18, 69.2% | 18, 72% | 1.00 |
| Program was fun | 41, 80.4% | 20, 76.9% | 21, 84% | 0.72 |
| Wanted to stop | 6, 11.8% | 3, 11.5% | 3, 12% | 1.00 |
| Was challenging enough | 42, 82.4% | 20, 76.9% | 22, 88% | 1.00 |
| Keep this exercise going after the program ends | 40, 78.4% | 22, 84.6% | 18, 72% | 0.324 |
| Looked forward to the exercise program | 42, 82.4% | 20, 76.9% | 22, 88% | 0.465 |
| Three sessions per week too much | 3, 5.9% | 2, 7,7% | 1, 4% | 1.00 |
| Exercise helped my mental health | 45, 88.2% | 22, 84.6% | 23, 92% | 0.67 |
| Wish I could have changed to a different type of exercise | 6, 13.7% | 2, 7.7% | 5, 20% | 0.25 |
| The program made positive changes to my body | 74.5% | 19, 73.1% | 19, 76% | 1.00 |
| Confident to do this on my own after it ends | 39, 76.5% | 20, 76.9% | 19, 76% | 1.00 |
| Individual sessions improved my mood | 44, 86.3% | 20, 76.9% | 24, 96% | **0.09** |

Bolded text indicates statistical significance.
